# Non-pharmacological therapy for chemotherapy-induced peripheral neurotoxicity: a network meta-analysis of randomized controlled trials

**DOI:** 10.1186/s12883-023-03485-z

**Published:** 2023-12-11

**Authors:** Xia Zhang, Ao Wang, Miaowei Wang, Guo Li, Quan Wei

**Affiliations:** 1 Rehabilitation Medicine Center, Institute of Rehabilitation Medicine, West China Hospital, Sichuan University, Chengdu, Sichuan People’s Republic of China; 2 Key Laboratory of Rehabilitation Medicine in Sichuan Province, Chengdu, Sichuan People’s Republic of China

**Keywords:** Chemotherapy, Adjuvant, Peripheral nervous system diseases, Randomized controlled trial

## Abstract

**Background:**

Chemotherapy-induced peripheral neurotoxicity (CIPN) is the most common adverse effect in patients undergoing chemotherapy, and no effective interventions are currently available for its prevention and treatment. Non-pharmacological therapies appear to be beneficial for the prevention and treatment of CIPN, but it remains unclear which therapy is most effective. The aim of this study was to identify the most effective non-pharmacological therapy for CIPN patients.

**Methods:**

PubMed, Web of Science, Embase, and Cochrane Library were searched for randomized controlled trials on non-pharmacological therapies for CIPN. The primary outcomes included pain and peripheral neuropathological symptoms, and the secondary outcomes included quality of life, sensory and motor symptoms. The pairwise analysis and a network meta-analysis were performed using a random effects model.

**Results:**

A total of 46 articles were included in this study, involving 2,878 participants. Our study showed that massage was more effective in pain-alleviating compared with acupuncture [SMD = 0.81, 95%CI (0.04, 1.57)], vitamin and gabapentin [SMD = 2.56, 95%CI (1.39, 3.74)], and usual care and placebo [SMD = 0.9, 95%CI (0.31, 1.49)]. As for attenuating peripheral neuropathological symptoms, massage was more effective than usual care and placebo [SMD = 0.75, 95%CI (0.33, 1.17)], sensorimotor training [SMD = 1.17, 95%CI (0.24, 2.10)], electrostimulation [SMD=-1.18, 95%CI (-2.14, -0.21)], multimodal exercise [SMD=-0.82, 95%CI (-1.57, -0.08)], and resistance training [SMD = 1.03, 95%CI (0.11, 1.95)]. Massage was also more effective than other non-pharmacological therapies in improving quality of life, sensory and motor symptoms.

**Conclusions:**

According to our study, massage has advantages in alleviating pain, improving quality of life, and improving peripheral neuropathological symptoms and has better effect than other non-pharmacological interventions, representing certain clinical significance. However, the results of this study should be interpreted with caution due to the limitations of the included studies. In the future, more high-quality multi arm randomized controlled trials can be attempted to provide direct comparisons of the relative effects of non-pharmacological interventions.

**Supplementary Information:**

The online version contains supplementary material available at 10.1186/s12883-023-03485-z.

## Introduction

Chemotherapy is one of the most commonly used anti-cancer treatments. Chemotherapy-induced peripheral neuropathy (CIPN) refers to a dose-limiting adverse effect that commonly occurs in patients receiving neurotoxic chemotherapies such as taxane, platinum agents, vinca alkaloids, thalidomide, and bortezomib. CIPN is clinically characterized by varying degrees of sensory, motor, and autonomic nervous dysfunction. Sensory symptoms occur firstly in the extremities as numbness, pain, or burning sensation, with a “sock-and-glove-like” distribution. Loss of vibration sense and joint position sense could be observed in severe cases, and it affects gait and balance, thereby exerting an adverse impact on patients’ quality of life [1]. The incidence of motor symptoms is lower than that of sensory symptoms. Motor symptoms typically included distal limb weakness, gait and balance dysfunction. Autonomic nervous dysfunction is characterized by orthostatic hypotension, constipation, sexual dysfunction, etc.

A review on CIPN has proposed that CIPN could occur in approximately 30-40% of patients receiving neurotoxic chemotherapy [2], and the effects of CIPN on the nervous system depend on the types of chemotherapeutic agents, pharmacological properties, and accumulated dose. A study has shown that 30% of CIPN patients could not recover from chemotherapy six months after chemotherapy [3].

The 2020 updated clinical practice guideline released by the American Society of Clinical Oncology (ASCO) indicates that there is currently no pharmacological agent capable of preventing CIPN, and evidence of moderate quality recommends duloxetine for patients with CIPN-associated pain after chemotherapy [4]. To date, the treatment of CIPN is still under exploration. Recent evidence has revealed that non-pharmacological therapies, such as exercise, acupuncture, pressure therapy, and scrambler therapy seem to be of potential benefits [4].

Previous studies have reported that acupuncture, massage, and pedilavium could alleviate the symptoms of CIPN [5]. Moreover, a meta-analysis on acupuncture indicates that acupuncture could effectively attenuate the pain and dysfunction in CIPN patients [6]. Another study has assessed the effect of exercise on peripheral neuropathological symptoms and reported that sensorimotor training could improve static balance, quality of life, and neuropathological symptoms in CIPN patients [7]. However, these studies focus on a single certain therapy for CIPN, lacking a systematical comparison among various non-pharmacological therapies.

The current study is the first network meta-analysis (NMA) to assess the comparative effects of non-pharmacological therapies for the treatment of CIPN. Through the NMA approach, we can summarize the direct and indirect evidence and perform pairwise comparisons between different interventions. This study has included randomized controlled trials (RCTs) that evaluate the efficacy of non-pharmacological interventions for CIPN patients, with other conventional treatments like placebo, or other non-pharmacological interventions as controls and pain, neuropathological symptoms, quality of life, sensory and motor symptoms as outcome measures. This study compares the direct and indirect evidence of various non-pharmacological interventions using a network meta-analysis and provides a ranking based on the comparative effect probability of each intervention to identify the most effective intervention, to provide evidence-based reference for clinical practice.

## Methods

This systematic review has been registered with PROSPERO (registration No.: CRD42022350831) and was conducted and reported in accordance with the PRISMA Extension Statement for Reporting of Systematic Reviews Incorporating Network Meta-analyses of Health Care Interventions (PRISMA-NMA) [8].

### Search strategy and study selection

We initially searched PubMed, Embase, Web of Science, and Cochrane Library up to May 9th, 2022, for RCTs on non-pharmacological therapies for CIPN patients. To avoid missing eligible literature, we searched the above-mentioned databases again up to October 22, 2023.

The search strategy exemplified by PubMed is as follows: ((“Chemotherapy, Adjuvant“[Mesh]) AND “Peripheral Nervous System Diseases“[Mesh]) AND (“Randomized Controlled Trial” [Publication Type] OR “Randomized Controlled Trials as Topic“[Mesh]). The detailed search strategy is shown in Supplementary material 1. Duplicates were removed using EndNote X9. Study selection was conducted by two reviewers (GL and AW) independently according to the inclusion and exclusion criteria. Disagreement was settled by discussion with a third reviewer (MW). If multiple articles were from the same study, the one with the latest publication date and more appropriate outcomes was included.

#### Inclusion criteria

- Patients had to have been diagnosed with CIPN.
- Non-pharmacological therapy had to be used as the interventions, such as exercise, acupuncture, cryotherapy, and electrostimulation.
- The study design must have been an RCT.
- The studies had to report at least one of the following outcome measures: pain, neuropathological symptoms, quality of life, and sensory and motor symptoms.
- The studies had to be reported and published in English.

#### Exclusion criteria

- Studies were excluded for the following reasons:
- Chemotherapeutic agents were used as interventions, despite the dosage and infusion speed.
- With a sample size of less than 20.
- Non-Clinical RCT design.
- Single-arm design.

### Outcomes

The primary outcomes included pain and peripheral neuropathological symptoms. Secondary outcomes included the quality of life, and sensory and motor symptoms. We only extracted data on pain, peripheral neuropathological symptoms, sensory and motor symptoms and failed to extract that on autonomic symptoms-related outcome measures due to that sensory nerve dysfunction is more common than the involvement of motor function [9], and autonomic symptoms were less common in CIPN patients [10]. Among the included studies, the pain assessment was completed using multiple scales including Numeric Rating Scale (NRS), Visual Analogue Scale (VAS), and Brief Pain Inventory (BPI). Peripheral neuropathological symptoms assessment was also completed using multiple scales such as Functional Assessment of Cancer Therapy/Gynecologic Oncology Group-Neurotoxicity subscale (FACT/GOG-Ntx subscale), Total Neuropathy Score (TNS), European Organization for Research and Treatment of Cancer Quality of Life-Chemotherapy-Induced Peripheral Neuropathy 20 (EORTC-QLQ-CIPN20), and Modified Total Neuropathy Score (mTNS), in which a higher score of FACT/GOG-Ntx subscale indicated more mild neuropathological symptoms whereas a higher score of other scales indicated the opposite. Quality of life assessment was completed using the European Organization for Research and Treatment of Cancer Quality of Life Core Questionnaire 30 (EORTC QLQ-C30), Functional Assessment of Cancer Therapy-Breast (FACT-B), and Functional Assessment of Cancer Therapy-General (FACT-G). Sensory and motor symptom assessment was based on EORTC QLQ-CIPN20. For a study using multiple tools to assess one certain outcome, we extracted the outcome data measured by the most commonly used tool.

### Data extraction

Data extracted mainly included characteristics of the study (name of the first author, publication year, title, study design, and setting), characteristics of participants (types of cancer, sample size, age, and gender), interventions (types and follow-up duration), and outcome measures (outcome data before and after treatment, and endpoint-baseline change calculated using mean and standard deviation). Missing data were obtained by contacting the authors. For several studies that only provided picture data rather than original data, the picture data were extracted using OriginPro2021 9.8.0.200 software. For several articles that were from the same study and reported the same results, only data from the most recently published article were extracted.

Several types of interventions were pooled due to too many non-pharmacological therapies included: (1) Acupuncture (Acu), (2) Massage, (3) Multimodal exercise (ME) that referred to the combination of more than two types of exercise or physical activities, (4) Education (Edu) such as nursing education and cognitive behavior therapy, (5) Electrostimulation (ES) such as low-frequency electric stimulation and scrambler therapy, and (6) Usual care or placebo (UCP). Other types that could not be classified into the above categories were listed as described, such as endurance training (ET), resistance training (RT), and whole body vibration therapy (WBV).

### Risk of bias assessment

The risk of bias was assessed according to the Cochrane Collaboration’s risk-of-bias tool [11], which contains seven domains: (1) random sequence generation, (2) allocation concealment, (3) blinding of participants and personnel, (4) blinding of outcome assessor, (5) incomplete data, (6) selective reporting, and (7) other sources of bias. Data extraction and quality assessment were conducted by two reviewers independently, and disagreements were settled through discussion with the third reviewer.

### Data synthesis and analysis

NMA within a Bayesian framework was performed using the STATA15.0 software to evaluate the effects of different interventions. Standardized mean difference (SMD) with 95% confidence interval (95%CI) was applied for effect size estimates of continuous variables, and risk ratio (RR) for dichotomous data [12, 13]. Given the diversity of the included interventions and the potential heterogeneity among the studies, a random effects model was used for meta-analysis. The inconsistency between direct and indirect evidence was assessed using node splitting analysis and inconsistency model (50,000 iterations, 20,000 annealing). When the *p*-value of node splitting analysis is greater than 0.05, a consistency model was used to estimate the ranking probabilities. If it was not practical, an inconsistency model was used. The surface under the cumulative ranking curve (SUCRA) and mean rank were applied to provide the cumulative ranking probabilities of each intervention. An intervention with the largest SUCRA value was the most effective in the network. If inconsistencies were found, the heterogeneity test was performed. The funnel plot was used to test publication bias.

## Results

### Characteristics of included studies

**Fig. 1:**
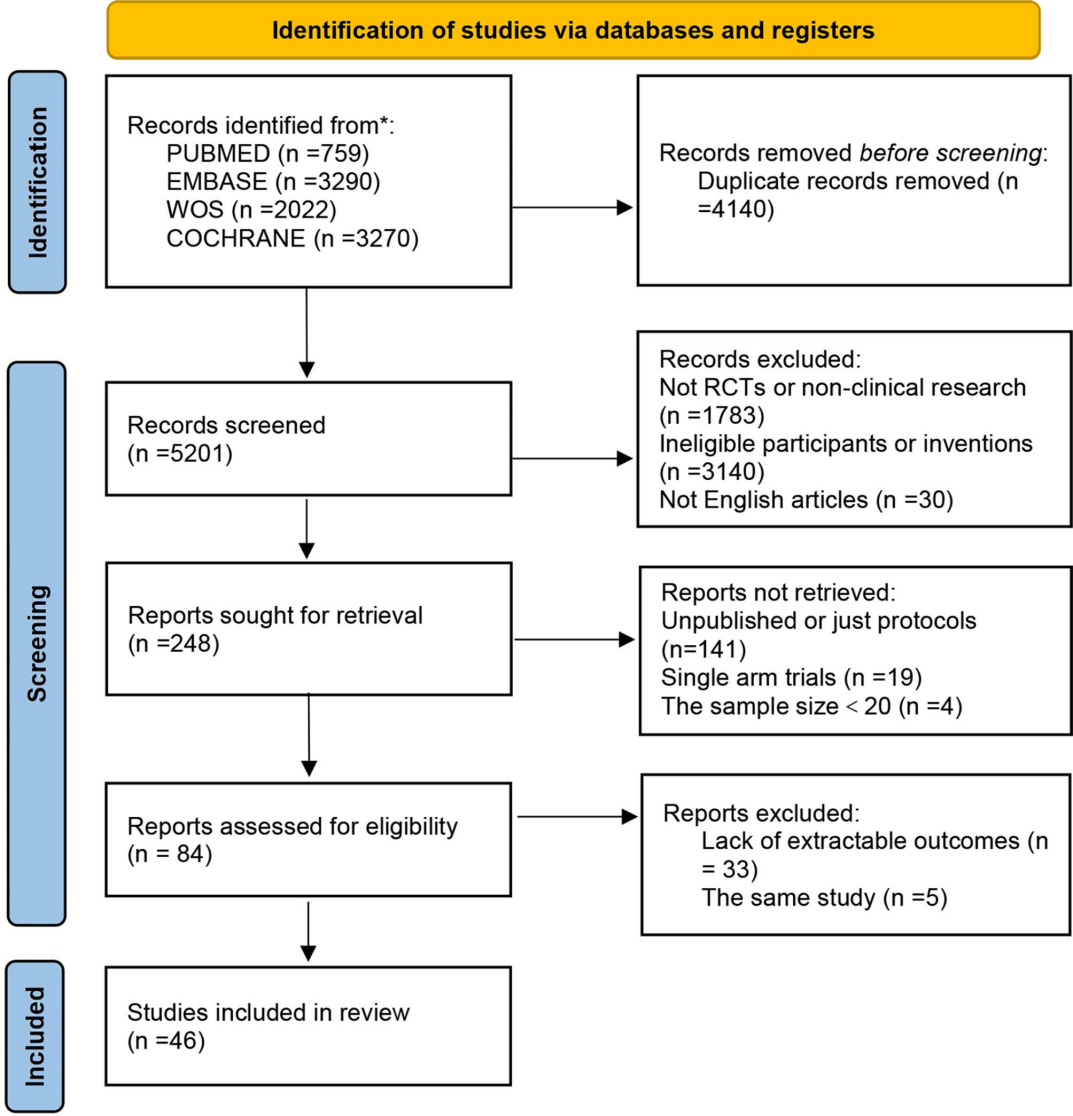
Flow chart of study selection and identification

According to the search strategy, a total of 9,341 records were obtained, and 4,140 duplicates were removed. According to the inclusion and exclusion criteria, a total of 84 articles were eligible, with 33 being excluded due to unavailable data and five being excluded due to overlapping publication. Finally, 45 studies (46 articles with 2,878participants were included for NMA, as shown in Fig. 1. Among the included studies, 42 studies examined the effects of two different interventions, two studies adopted a three-arm design, and one study adopted a four-arm design. A list and detailed characteristics of the included studies are provided in Supplementary material 2.

### Risk of bias assessment

**Fig. 2:**
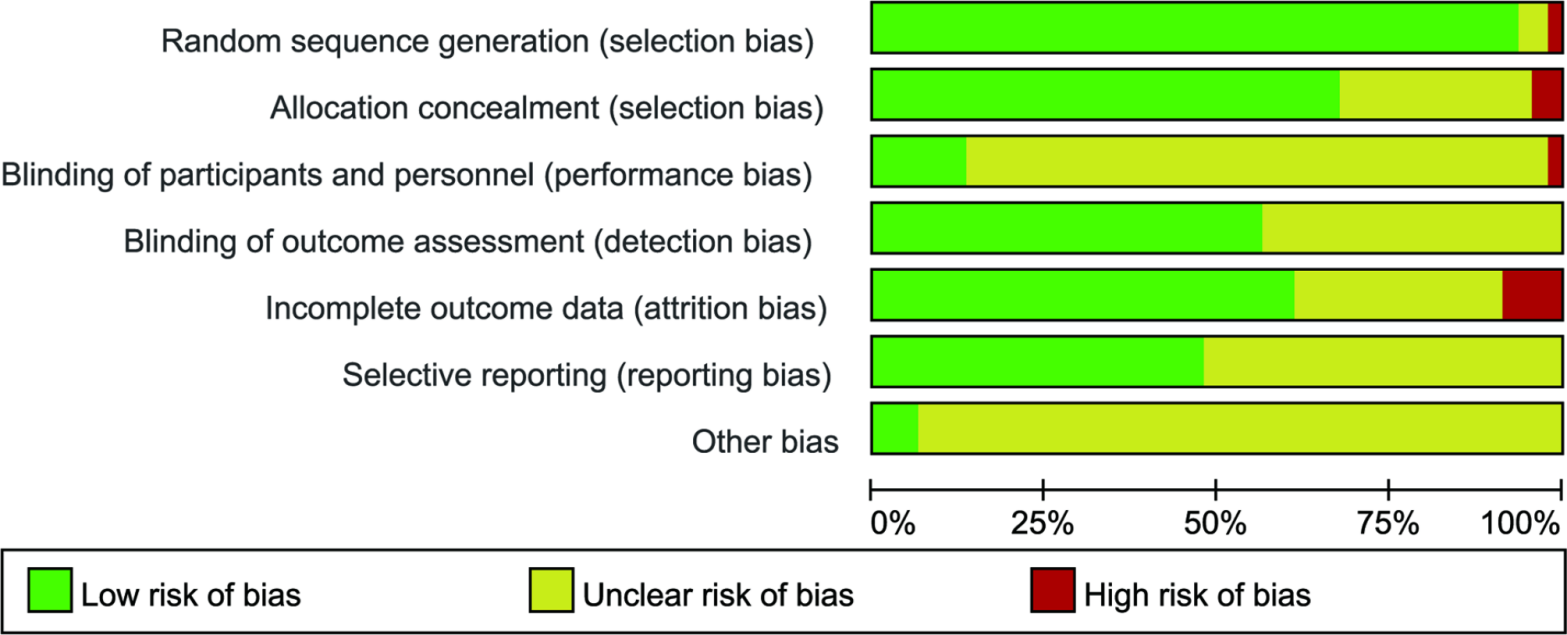
Risk of bias graph. Review authors’ judgments about each risk of bias item across all the included studies are presented as percentages. The green, yellow, and red colors represent the low risk of bias, unclear risk of bias, and high risk of bias, respectively

**Fig. 3:**
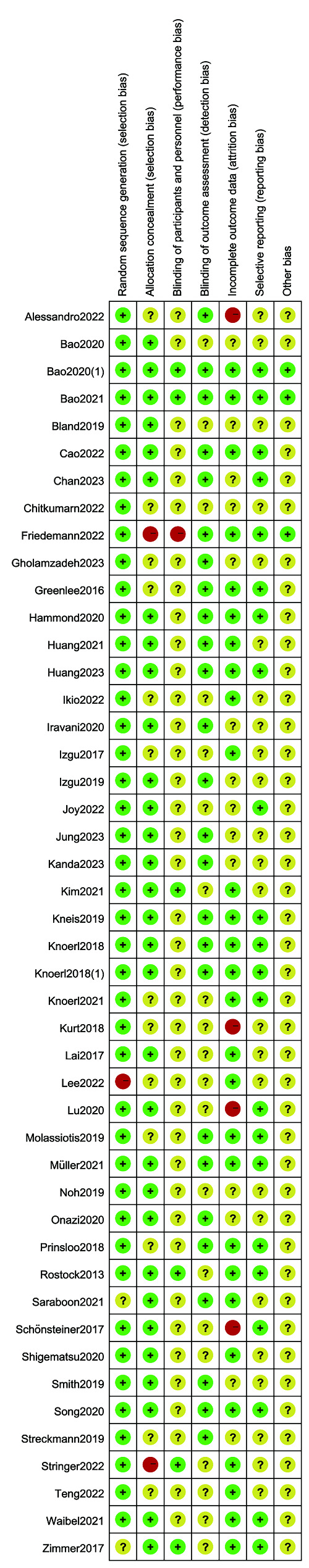
Risk of bias summary. Review authors’ judgments about each risk of bias item

The ratio of studies with a low, moderate, and high risk of bias for the individual items was as follows: randomization sequence generation (93.5%, 4.3%, and 2.2%, respectively), allocation concealment (69.6%, 28.2%, and 4.3%, respectively), blinding of participants and personnel (13%, 84.8%, and 2.2%, respectively), blinding of outcome assessor (56.5%, 43.5%, and 0%, respectively), incomplete data (60.9%, 30.4%, and 8.7%, respectively), selective reporting (47.9%, 52.1%, and 0%, respectively), and other sources of bias (6.5%, 93.5%, and 0%, respectively). Detailed information about risk of bias assessment for the included studies are shown in Figs. 2 and 3.

### Primary outcomes

#### Pain

**Fig. 4:**
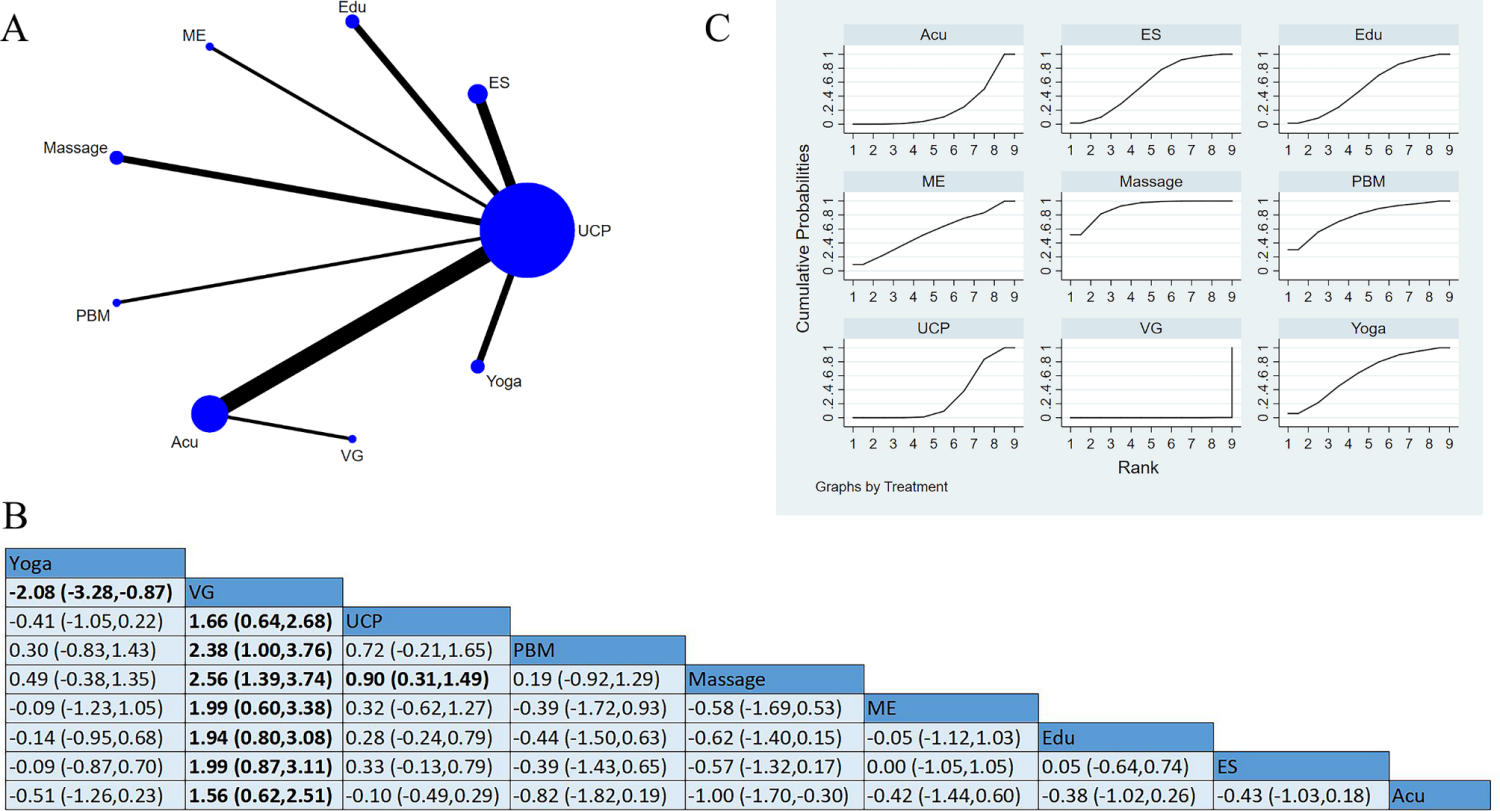
Pain: (**A**) Network meta-analysis of eligible comparisons; (**B**) Cumulative ranking probability plots. Each node represents an intervention, and the connecting lines between two nodes represent one or more randomized clinical trials (RCTs) in which the two interventions have been compared directly. The size of each node is proportional to the number of randomly assigned participants, and the thickness of the lines connecting two nodes is weighted according to the number of RCTs that directly compared the two specific interventions they connect. The horizontal axis represents the possible rank of each treatment (from best to worst according to the outcome). The vertical axis represents the cumulative probability for each treatment to be the best option, the best of 2 options, the best of 3 options, and so on. Higher SUCRA indicates more excellent therapeutic effect; (**C**) Network meta-analysis of effectiveness

Seventeen studies reported pain-alleviating, involving 956 participants [14–30]. Among these studies, three studies applied ES as an intervention, including scrambler therapy [14], low-frequency electrical stimulation [16], and neurofeedback therapy [17], 16 studies focused on UCP, two studies adopted interventions pooled as Yoga [15, 18], two studies used massage [19, 27], two studies used interventions pooled as Edu, including cognitive behavioral therapy [20] and electronic symptom management system [22], six studies used Acu [23–26, 29, 30], one used vitamin and gabapentin (VG [24]), one used photobiomodulation (PBM [21]), and one used ME [28]. There were statistical differences between massage and Acu, VG, and UCP. Massage presented to be more effective than Acu [SMD=-1.0, 95%CI (-1.70, -0.30)], VG [SMD = 2.56, 95%CI (1.39, 3.74)], and UCP [SMD = 0.9, 95%CI (0.31, 1.49)]. Yoga was more effective than VG [SMD=-2.08, 95%CI (-3.28, -0.87)]. SUCRA based on the accumulative probability ranking showed that the top three interventions with the SUCRA values were massage (90.4%), PBM (77.2%), and Yoga (62.8). Massage had the highest probability of being the most effective intervention in pain-alleviating (Fig. 4).

#### Peripheral neuropathological symptoms

##### *Peripheral neuropathological symptoms- FACT/GOG-Ntx subscale*

**Fig. 5:**
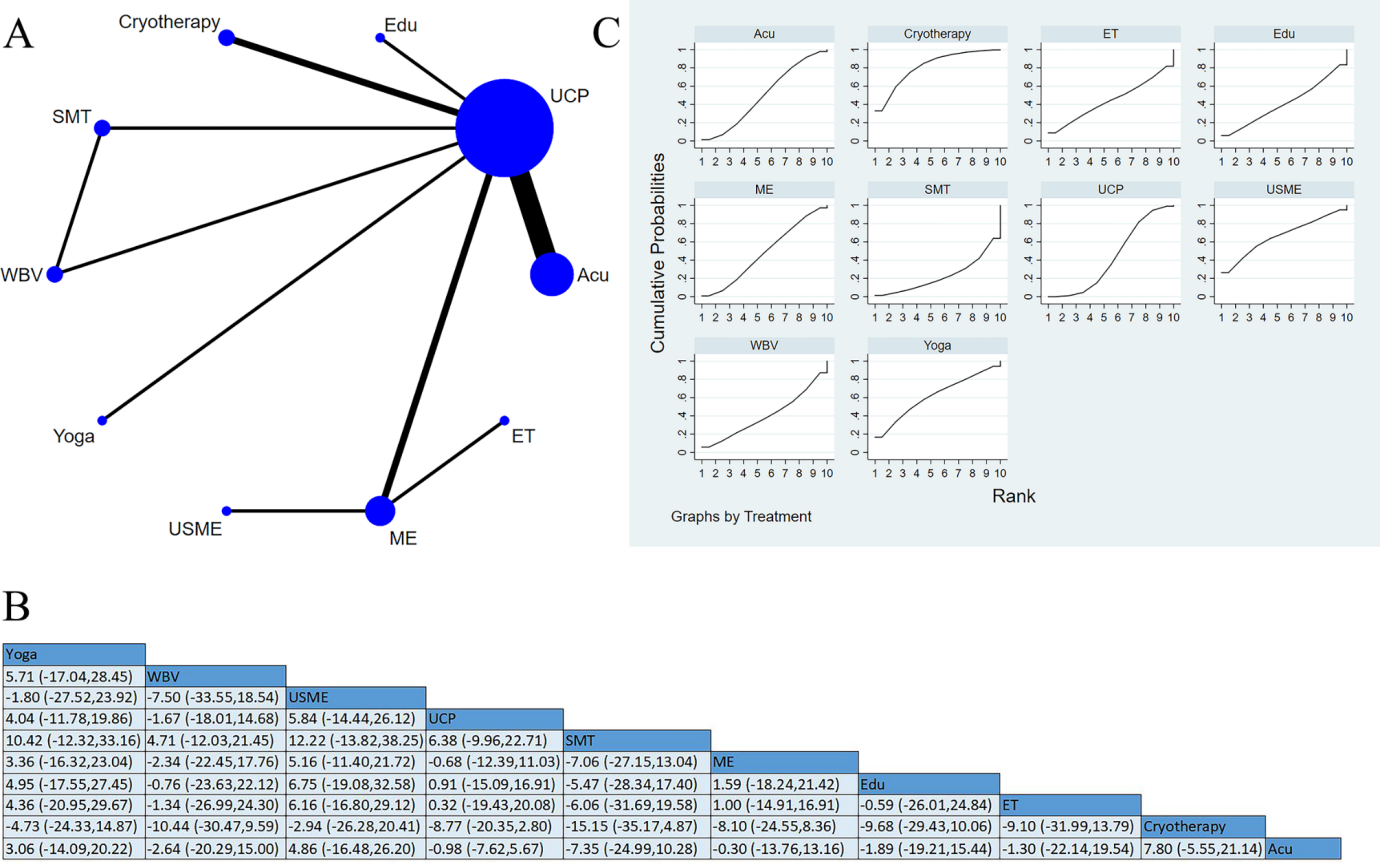
Peripheral neuropathological symptoms-FACT/GOG-Ntx: (**A**) Network meta-analysis of eligible comparisons; (**B**) Cumulative ranking probability plots; (**C**) Network meta-analysis of effectiveness

Twenty-five studies reported peripheral neuropathological symptoms. Subgroup analysis was performed by different scales. Among the studies, 15 applied the FACT/GOG-Ntx subscale for assessment [15, 25, 28, 30–41], involving 667 participants. A higher score of this subscale indicated more mild neuropathological symptoms. Additionally, four studies used interventions pooled as ME [28, 33, 34, 37], and 13 studies used interventions pooled as UCP. One three-arm trial [31] conducted a direct comparison of sensorimotor training (SMT), WBV, and UCP, two used cryotherapy [32, 38], six used Acu [25, 30, 35, 36, 39, 40] and one used ultrasound and multicomponent exercise (USME [34]). The close loop of the network map was formed by SMT, WBV, and UCP. Pairwise comparison showed no statistical difference between different interventions, but SUCRA based on the accumulative probability ranking showed that the top three interventions were cryotherapy (81.5%), USME (66.5%), and Yoga (61.9%). (Fig. 5).

##### *Peripheral neuropathological symptoms-other scales*

**Fig. 6:**
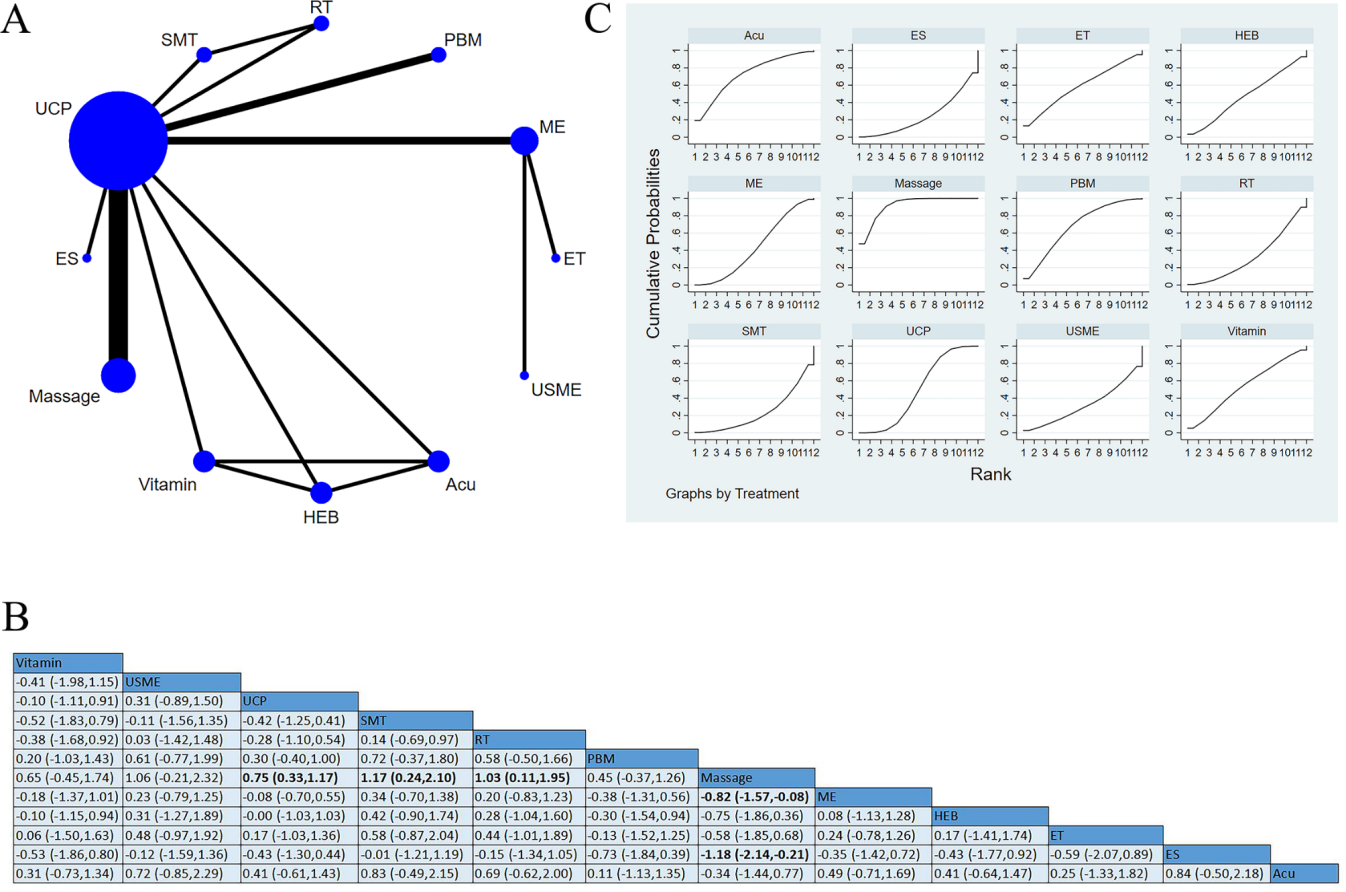
Peripheral neuropathological symptoms-other scales: (**A**) Network meta-analysis of eligible comparisons; (**B**) Cumulative ranking probability plots; (**C**) Network meta-analysis of effectiveness

Fourteen studies applied other scales for assessment [14, 21, 27, 34, 42–51], involving 857 participants. One four-arm trial [43] conducted direct a comparison among Acu, hydroelectric baths (HEB), vitamin, and UCP, one study used ES, one three-arm study conducted a direct comparison among SMT, RT, and UCP [44], five studies used massage [27, 42, 45, 46, 51], four study used ME [34, 47, 48, 50], and one study used PBM [21]. A close loop of the network map was formed by SMT, RT, and UCP. Moreover, Acu, HEB, vitamin, and UCP also formed a close loop. Massage presented to be more effective in alleviating neuropathological symptoms, compared with UCP [SMD = 0.75, 95%CI (0.33, 1.17)], SMT [SMD = 1.17, 95%CI (0.24, 2.10)], and RT [SMD = 1.03, 95%CI (0.11, 1.95)]. NMA showed that the interventions with the top three comparative effects were massage (91.9%), Acu (72.6%), and PBM (67.9%). The results showed that massage could contribute to improving neuropathological symptoms (Fig. 6).

### Secondary outcomes

#### Quality of life

**Fig. 7:**
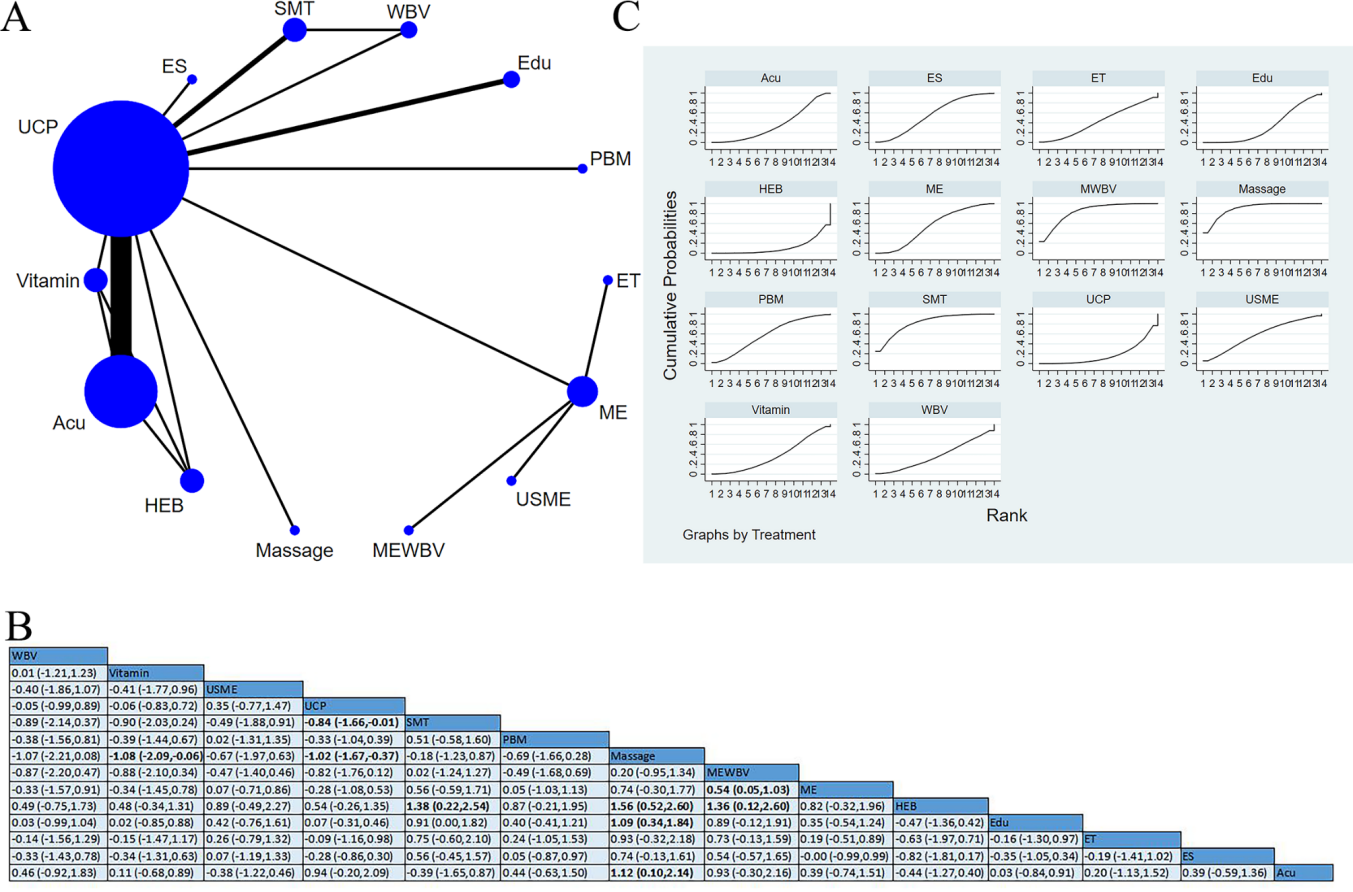
Quality of life: (**A**) Network meta-analysis of eligible comparisons; (**B**) Cumulative ranking probability plots; (**C**) Network meta-analysis of effectiveness

Nineteen studies [14, 25, 26, 30, 31, 33–37, 41–43, 49, 52–56] that reported quality of life, involving 1,063 participants. Among these studies, eight studies applied Acu [25, 26, 30, 35, 36, 43, 53, 56], two studies used interventions pooled as SMT [31, 55], one study used ES [14], four studies used ME [33, 34, 37, 52], one study used multicomponent exercise and whole vibration training (MEWBV [52]). One four-arm trial [43] conducted a direct comparison among Acu, HEB, vitamin, and UCP, and one three-arm study conducted a direct comparison among SMT, RT, and UCP [31]. A close loop of the network map was formed by Acu, HEB, vitamin, and UCP. Another close loop was formed by SMT, WBV, and UCP. Massage presented to be significantly more effective than HEB [SMD = 1.56, 95%CI (0.52, 2.60)], UCP [SMD=-1.02, 95%CI (-1.67, -0.37)], Acu [SMD = 1.12, 95%CI (0.10, 2.14)] and Edu [SMD = 1.09, 95%CI (0.34, 1.84)]. SMT was significantly more effective than HEB [SMD = 1.38, 95%CI (0.22, 2.54)] and UCP [SMD=-0.84, 95%CI (-1.66, -0.01)]. MEWBV was more effective than ME [SMD = 0.54, 95%CI (0.05, 1.03)] and HEB [SMD = 1.36, 95%CI (0.12, 2.60)]. SUCRA based on the accumulative probability ranking showed that the top three interventions were massage (90.3%), MEWBV (84.1%), and SMT (82.8%). (Fig. 7).

#### Sensory symptoms

**Fig. 8:**
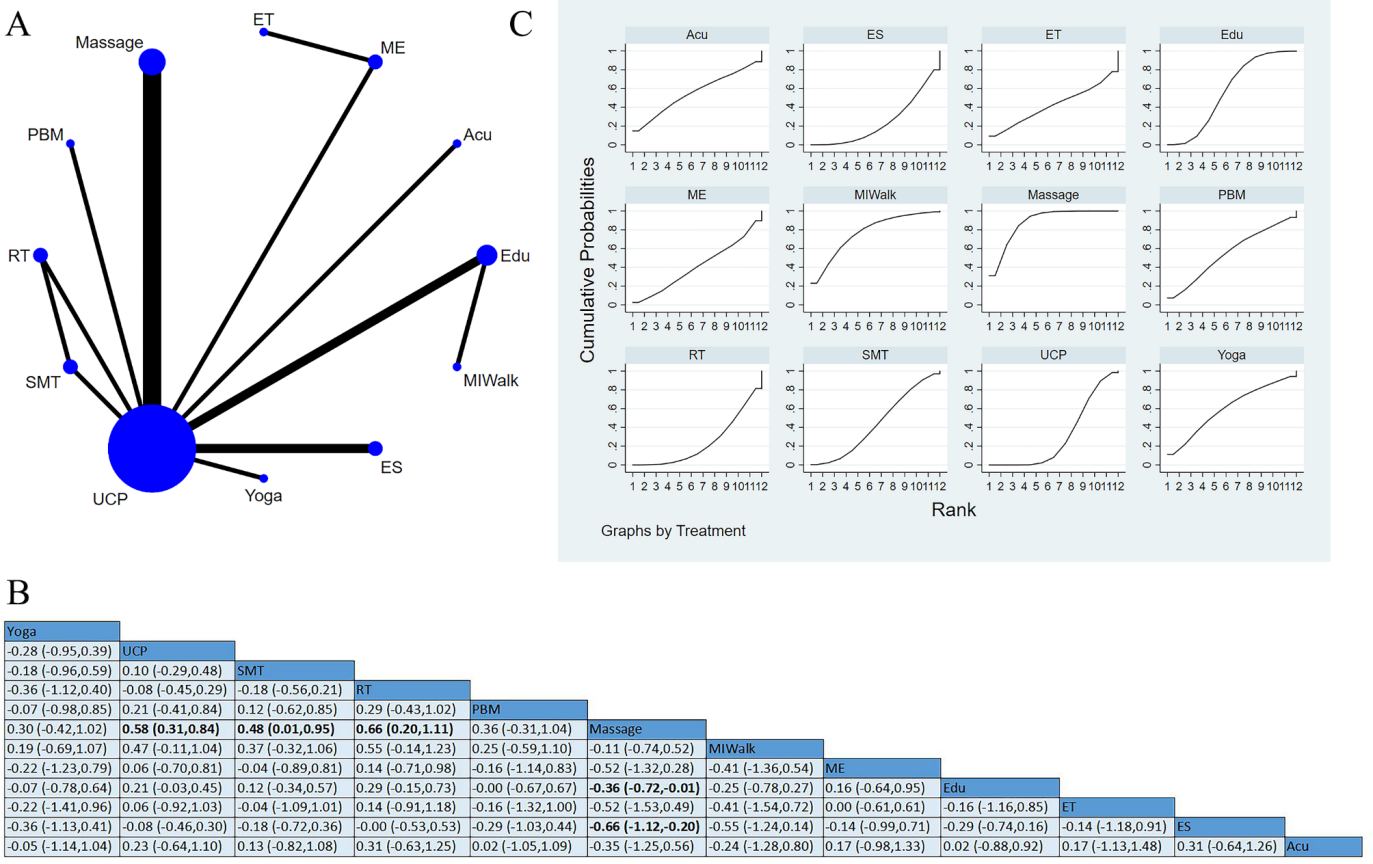
Sensory symptoms: (**A**) Network meta-analysis of eligible comparisons; (**B**) Cumulative ranking probability plots; (**C**) Network meta-analysis of effectiveness

Fifteen studies [14, 16, 18–20, 22, 26, 37, 44, 46, 49, 51, 57–59] reported sensory symptoms, involving 991 participants. Among the studies, two studies used interventions pooled as ES [14, 16], 13 studies used UCP, two applied ME [37, 57], four used studies pooled as massage [19, 46, 51, 58], 3 applied Edu [20, 22, 59]. One three-arm trial conducted a direct comparison among SMT, RT, and UCP [44], and one study applied ET [37]. One study conducted a direct comparison between Edu and Motivational Interviewing-Walk (MIWalk [59]), one study used Yoga [18], and one used Acu [26]. Massage presented to be more effective than UCP [SMD = 0.58, 95%CI (0.31, 0.84)], SMT [SMD = 0.48, 95%CI (0.01, 0.95)], Edu [SMD=-0.36, 95%CI (-0.72, -0.01)], ES [SMD=-0.66, 95%CI (-1.12, -0.2)] and RT [SMD = 0.66, 95%CI (0.20, 1.11)]. The top three interventions were massage, MIWalk, and Yoga, and their SUCRA values were 88.3%, 77.1%, and 60.4%, respectively. The results showed that massage might be the most effective intervention in improving sensory symptoms in CIPN patients, as shown in Fig. 8.

#### Motor symptoms

**Fig. 9:**
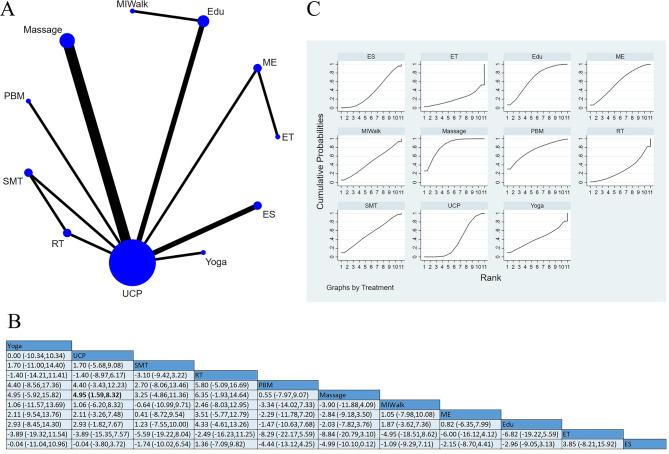
Motor symptoms: (**A**) Network meta-analysis of eligible comparisons; (**B**) Cumulative ranking probability plots. (**C**) Network meta-analysis of effectiveness

Fourteen studies [14, 16, 18–20, 22, 27, 37, 44, 46, 51, 57–59] reported motor symptoms, involving 970 participants. Among these studies, two applied ES [14, 16], two used interventions pooled as ME [37, 57], one conducted a direct comparison between ME and ET [37], four used massage [19, 46, 51, 58], three used interventions pooled as Edu [20, 22, 59], one three-arm trial [44] conducted a direct comparison among SMT, RT, and UCP, one study used Yoga, one study used PBM, and one used MIWalk. There was no direct comparison of MIWalk and ET with controls. There were significantly statistical differences between massage and UCP [SMD = 4.95, 95%CI (1.59, 8.32)]. The comparative effect probability ranking showed that the top three interventions were massage (84.0%), PBM (73.5%), and Edu (66.3%). The results showed that massage might be the most effective intervention in improving motor symptoms, as shown in Fig. 9.

### Inconsistency, heterogeneity analyses, and publication bias

The results of the primary outcomes and secondary outcomes for global inconsistency showed no significant global inconsistency. Node splitting analysis showed consistency between direct effects and indirect effects. The *p* values between the direct effects and indirect effects were all greater than 0.05. Therefore, we used a consistency model to estimate the ranking probabilities. There was no significant heterogeneity among studies. We used Egger’s test and funnel plot to assess the potential publication bias of the included studies. When the peripheral neuropathological symptoms were assessed using the FACT/GOG-Ntx subscale, there was publication bias among the studies, and the *P* value from Egger’ test was 0.009. The *P* values of other studies were greater than 0. 05, indicating that there was no obvious publication bias for these studies. (Figure S1)

## Discussion

According to the results of NMA, massage could be the most effective non-pharmacological intervention, especially in alleviating pain and improving neuropathological symptoms, and sensory and motor symptoms. A previous study has confirmed the effect of foot massage as an alternative therapy for diabetes-related peripheral neuropathological symptoms [60]. According to the National Comprehensive Cancer Network (NCCN) Clinical Practice Guidelines, massage has been an adjuvant therapy for the management of pain. However, its application remains controversial due to the methodological defects of relevant studies, such as limited sample size, improper control, and the uncontrolled placebo effect, as well as the lack of relevant mechanisms and high-quality research. The types of massage in this study included acupressure, reflexology massage, and classical massage. We found that massage could relieve the symptoms of CIPN, compared with the control group. The mechanism by which massage can relieve pain is related to the Gate Theory of Pain, the promotion of parasympathetic nerve activity, and the influence on body chemistry, such as serotonin and endorphin. At the same time, massage has mechanical effects, which can loosen adhesions, prevent fibrosis, and promote blood and lymph circulation. Massage is also related to the promotion of restorative sleep and interpersonal attention [61]. A case report that applied an infrared thermosensitive test to monitor superficial skin temperature showed that limb stiffness, sensory dysfunction, and pain were significantly alleviated after six weeks of massage in CIPN patients. The increase in superficial temperature suggested that the improvement of CIPN symptoms might be associated with changes in blood circulation [62]. This is the first report indicating that massage improves CIPN, while it lacks objective measurements such as nerve conduction study (NCS). Newly emerged RCTs using NCS combined with patient-reported results confirmed the efficacy of classical massage in breast cancer patients receiving taxane-related chemotherapy. Compared with the control group, classical massage could alleviate peripheral neuropathic pain, and results in a higher amplitude of sensory action potential in the median nerve and significantly shorter latency in the tibial nerve [46]. Kurt et al. have found that reflexology massage therapy fails to improve the physical activity level, motor function, and autonomic nerve-associated symptoms, while it effectively alleviates the sensory symptoms in CIPN patients [19].

Our study indicates that massage may be more effective in pain-alleviating than acupuncture. Although a previous study demonstrates that acupuncture is of neuropathy-improving effect in CIPN patients [36], the evidence is still insufficient. A study by GreenLee et al. indicates that acupuncture has unsatisfactory effect, compared with the sham-acupuncture control [25]. Another four-arm trial has yielded inconclusive results for the effect of acupuncture [31]. This may be related to the selection of different evaluation indicators. Since pain perception is subjective, massage may have an advantage in the improvement of subjective symptoms. In contrast, Acu may cause pain and discomfort during the treatment, and this may affect the subjective assessment of a patient despite a certain follow-up duration. There may be benefits for Acu using NCS for evaluation, but unfortunately, there are fewer studies using NCS for CIPN evaluation. Moreover, according to our study, although PBM and Yoga were not statistically significant compared with other interventions, SUCRA ranking indicated that they might have potential advantages. Previous studies indicate that acupuncture could alleviate CIPN-related pain and neuropathological symptoms [6]. However, with newly published studies added to our study, the subgroup analysis by the FACT/GOG-Ntx subscale fails to confirm the merit of acupuncture, when compared with other non-pharmacological interventions. According to the results in paragraph Peripheral neuropathological symptoms-other, massage was significantly more effective than SMT and RT, and this may be because that SMT is more likely to improve motor function, and RT tends to improve muscle strength. Nevertheless, these indicators were not used for the evaluation in this study.

As for the quality of life-improving, our study has found that massage is evidently more effective than Acu, Edu, and HEB, and SMT is more effective than HEB. This may be related to the heterogeneity of the results among Acu-related studies. Besides, there are only two studies related to Edu, and the results of these studies indicated no impact on quality of life.

As for sensory symptoms, ES did not show an advantage, with both ES-related studies included in the analysis having negative results. The pairwise comparison of massage and ES showed a significant difference.

Our study is based on 45 studies, involving 46 articles and 2,878 participants, and is the first NMA to assess the efficacy of non-pharmacological interventions for CIPN patients. Not limited to a single certain intervention, we aimed to explore the comparative effects of different types of non-pharmacological interventions for treating CIPN-induced pain, peripheral neuropathological symptoms, sensory and motor symptoms, and quality of life. Multiple non-pharmacological interventions were included in our study, such as acupuncture, cryotherapy, scrambler therapy, neurofeedback, water bath, photobiomodulation, yoga, exercise, and acupoint massage, and the results have revealed the benefits of non-pharmacological intervention in treating CIPN.

Our study has some limitations that should be addressed. First, most of the included studies are single-center trials, and few studies included were of high quality, with no long-term follow-up. Second, there is currently no standardized method available to assess chemotherapy-induced neurotoxicity [9], and the assessment tools vary in each study. In addition, most of the outcome measurement tools in the included studies are subjective scales, lacking objective measures. Patients may be affected by the blind method and placebo effects. Therefore, more studies using objective assessments are needed to further validate our findings. Third, the assessment indicators we selected are not comprehensive and only cover some CIPN-induced symptoms. Therefore, we cannot fully understand the effect of some interventions. Potential selective reporting bias may exist. Fourth, only a few of the included studies are multi-arm trials, and a few closed loops were formed in our network meta-analysis. Most of the interventions are compared indirectly rather than directly. Fifth, since there are numerous non-pharmacological interventions, they were categorized into several groups. Moreover, the studies related to some special types of intervention are limited in number, although they have been included in the NMA, and more research data are needed for further validation. In addition, only seven massage-related studies have been included in our NMA [19, 27, 42, 51, 55, 58, 60], including acupressure, reflexology massage, and classical massage. Although the results suggest that massage has advantages over other non-pharmacological therapies for CIPN patients, unfortunately, very few studies have directly compared other non-pharmacological interventions with massage.

## Conclusion

Our network meta-analysis has summarized the previous evidence by comparing the therapeutic effect of various non-pharmacological interventions for CIPN treatment. The analysis results indicate that massage may be the most effective non-pharmacological intervention in alleviating pain, neuropathological symptoms, and sensory and motor dysfunction in CIPN patients. Given the above-mentioned limitations and heterogeneity, the findings here should be interpreted with caution. In the future, more high-quality RCTs with large sample sizes should be conducted to further validate our findings. In addition, assessment tools with higher reliability and validity should be applied, in combination with clinical examination, objective neurophysiological parameters, and patient-reporting outcomes.

### Electronic supplementary material

Below is the link to the electronic supplementary material.

Supplementary Material 1

Supplementary Material 2

Supplementary Material 3

## Data Availability

Data sharing is not applicable to this article as no datasets were generated or analysed during the current study.

## Abbreviations

CIPN: Chemotherapy-induced peripheral neurotoxicity
ASCO: American Society of Clinical Oncology
NMA: Network meta-analysis
RCTs: Randomized controlled trials
NRS: Numeric Rating Scale
VAS: Visual Analogue Scale
BPI: Brief Pain Inventory
TNS: Total Neuropathy Score
mTNS: Modified Total Neuropathy Score
FACT-B: Functional Assessment of Cancer Therapy-Breast
FACT-G: Functional Assessment of Cancer Therapy-General
95%CI: 95% confidence interval
RR: Risk ratio
SUCRA: Surface under the cumulative ranking curve
VG: Vitamin and gabapentin
PBM: Photobiomodulation
MIWalk: Motivational interviewing walk
NCCN: National Comprehensive Cancer Network
NCS: Nerve conduction study
