# Supplementary material for: Non-pharmacological therapy for chemotherapy-induced peripheral neurotoxicity: a network meta-analysis of randomized controlled trials
Source: BMC Neurol. 2023 Dec 11;23:433. doi: 10.1186/s12883-023-03485-z (PMC10712106; doi:10.1186/s12883-023-03485-z)
Supplement: Supplementary file 1 — Supplementary Material 1 [file 12883_2023_3485_MOESM1_ESM.docx]

**Supplementary material 1: Search Strategy for Each Database**

| PUBMED: | |
| --- | --- |
| 14 | (((((((((((Randomized Controlled Trials as Topic[Title/Abstract]) OR (randomized controlled trial[Title/Abstract])) OR (Clinical Trials, Randomized[Title/Abstract])) OR (Trials, Randomized Clinical[Title/Abstract])) OR (Controlled Clinical Trials, Randomized[Title/Abstract])) OR (pragmatic clinical trials as topic[Title/Abstract])) OR (controlled clinical trial[Title/Abstract])) OR (randomized[Title/Abstract])) OR (randomly[Title/Abstract])) OR (blind[Title/Abstract])) OR (("Randomized Controlled Trial" [Publication Type]) OR "Randomized Controlled Trials as Topic"[Mesh])) AND ((((chemotherapy-induced peripheral neuropathy[Title/Abstract]) OR (chemotherapy neuropathy[Title/Abstract])) OR (chemotherapy adj3 neuropathy[Title/Abstract])) OR ((("Peripheral Nervous System Diseases"[Mesh]) OR ((((((((((((((((Peripheral Nervous System Diseases[Title/Abstract]) OR (Peripheral Nervous System Disease[Title/Abstract])) OR (PNS Diseases[Title/Abstract])) OR (PNS Disease[Title/Abstract])) OR (Peripheral Neuropathies[Title/Abstract])) OR (Neuropathy, Peripheral[Title/Abstract])) OR (Peripheral Neuropathy[Title/Abstract])) OR (Peripheral Nerve Diseases[Title/Abstract])) OR (Nerve Disease, Peripheral[Title/Abstract])) OR (Nerve Diseases, Peripheral[Title/Abstract])) OR (Peripheral Nerve Disease[Title/Abstract])) OR (Peripheral Nervous System Disorders[Title/Abstract])) OR (peripheral nerve disorder[Title/Abstract])) OR (peripheral nerve injuries[Title/Abstract])) OR (peripheral nervous disease[Title/Abstract])) OR (Polyneuropathy[Title/Abstract]))) AND ((("Chemotherapy, Adjuvant"[Mesh]) OR (((((Chemotherapy, Adjuvant[Title/Abstract]) OR (Chemotherapy[Title/Abstract])) OR (Drug Therapy, Adjuvant[Title/Abstract])) OR (Adjuvant Chemotherapy[Title/Abstract])) OR (Adjuvant Drug Therapy[Title/Abstract]))) OR (chemotherapeutics[Title/Abstract])))) |
| 13 | ((((((((((Randomized Controlled Trials as Topic[Title/Abstract]) OR (randomized controlled trial[Title/Abstract])) OR (Clinical Trials, Randomized[Title/Abstract])) OR (Trials, Randomized Clinical[Title/Abstract])) OR (Controlled Clinical Trials, Randomized[Title/Abstract])) OR (pragmatic clinical trials as topic[Title/Abstract])) OR (controlled clinical trial[Title/Abstract])) OR (randomized[Title/Abstract])) OR (randomly[Title/Abstract])) OR (blind[Title/Abstract])) OR (("Randomized Controlled Trial" [Publication Type]) OR "Randomized Controlled Trials as Topic"[Mesh]) |
| 12 | (((((((((Randomized Controlled Trials as Topic[Title/Abstract]) OR (randomized controlled trial[Title/Abstract])) OR (Clinical Trials, Randomized[Title/Abstract])) OR (Trials, Randomized Clinical[Title/Abstract])) OR (Controlled Clinical Trials, Randomized[Title/Abstract])) OR (pragmatic clinical trials as topic[Title/Abstract])) OR (controlled clinical trial[Title/Abstract])) OR (randomized[Title/Abstract])) OR (randomly[Title/Abstract])) OR (blind[Title/Abstract]) |
| 11 | (((chemotherapy-induced peripheral neuropathy[Title/Abstract]) OR (chemotherapy neuropathy[Title/Abstract])) OR (chemotherapy adj3 neuropathy[Title/Abstract])) OR ((("Peripheral Nervous System Diseases"[Mesh]) OR ((((((((((((((((Peripheral Nervous System Diseases[Title/Abstract]) OR (Peripheral Nervous System Disease[Title/Abstract])) OR (PNS Diseases[Title/Abstract])) OR (PNS Disease[Title/Abstract])) OR (Peripheral Neuropathies[Title/Abstract])) OR (Neuropathy, Peripheral[Title/Abstract])) OR (Peripheral Neuropathy[Title/Abstract])) OR (Peripheral Nerve Diseases[Title/Abstract])) OR (Nerve Disease, Peripheral[Title/Abstract])) OR (Nerve Diseases, Peripheral[Title/Abstract])) OR (Peripheral Nerve Disease[Title/Abstract])) OR (Peripheral Nervous System Disorders[Title/Abstract])) OR (peripheral nerve disorder[Title/Abstract])) OR (peripheral nerve injuries[Title/Abstract])) OR (peripheral nervous disease[Title/Abstract])) OR (Polyneuropathy[Title/Abstract]))) AND ((("Chemotherapy, Adjuvant"[Mesh]) OR (((((Chemotherapy, Adjuvant[Title/Abstract]) OR (Chemotherapy[Title/Abstract])) OR (Drug Therapy, Adjuvant[Title/Abstract])) OR (Adjuvant Chemotherapy[Title/Abstract])) OR (Adjuvant Drug Therapy[Title/Abstract]))) OR (chemotherapeutics[Title/Abstract]))) |
| 10 | ((chemotherapy-induced peripheral neuropathy[Title/Abstract]) OR (chemotherapy neuropathy[Title/Abstract])) OR (chemotherapy adj3 neuropathy[Title/Abstract]) |
| 9 | (("Peripheral Nervous System Diseases"[Mesh]) OR ((((((((((((((((Peripheral Nervous System Diseases[Title/Abstract]) OR (Peripheral Nervous System Disease[Title/Abstract])) OR (PNS Diseases[Title/Abstract])) OR (PNS Disease[Title/Abstract])) OR (Peripheral Neuropathies[Title/Abstract])) OR (Neuropathy, Peripheral[Title/Abstract])) OR (Peripheral Neuropathy[Title/Abstract])) OR (Peripheral Nerve Diseases[Title/Abstract])) OR (Nerve Disease, Peripheral[Title/Abstract])) OR (Nerve Diseases, Peripheral[Title/Abstract])) OR (Peripheral Nerve Disease[Title/Abstract])) OR (Peripheral Nervous System Disorders[Title/Abstract])) OR (peripheral nerve disorder[Title/Abstract])) OR (peripheral nerve injuries[Title/Abstract])) OR (peripheral nervous disease[Title/Abstract])) OR (Polyneuropathy[Title/Abstract]))) AND ((("Chemotherapy, Adjuvant"[Mesh]) OR (((((Chemotherapy, Adjuvant[Title/Abstract]) OR (Chemotherapy[Title/Abstract])) OR (Drug Therapy, Adjuvant[Title/Abstract])) OR (Adjuvant Chemotherapy[Title/Abstract])) OR (Adjuvant Drug Therapy[Title/Abstract]))) OR (chemotherapeutics[Title/Abstract])) |
| 8 | ("Peripheral Nervous System Diseases"[Mesh]) OR ((((((((((((((((Peripheral Nervous System Diseases[Title/Abstract]) OR (Peripheral Nervous System Disease[Title/Abstract])) OR (PNS Diseases[Title/Abstract])) OR (PNS Disease[Title/Abstract])) OR (Peripheral Neuropathies[Title/Abstract])) OR (Neuropathy, Peripheral[Title/Abstract])) OR (Peripheral Neuropathy[Title/Abstract])) OR (Peripheral Nerve Diseases[Title/Abstract])) OR (Nerve Disease, Peripheral[Title/Abstract])) OR (Nerve Diseases, Peripheral[Title/Abstract])) OR (Peripheral Nerve Disease[Title/Abstract])) OR (Peripheral Nervous System Disorders[Title/Abstract])) OR (peripheral nerve disorder[Title/Abstract])) OR (peripheral nerve injuries[Title/Abstract])) OR (peripheral nervous disease[Title/Abstract])) OR (Polyneuropathy[Title/Abstract])) |
| 7 | (("Chemotherapy, Adjuvant"[Mesh]) OR (((((Chemotherapy, Adjuvant[Title/Abstract]) OR (Chemotherapy[Title/Abstract])) OR (Drug Therapy, Adjuvant[Title/Abstract])) OR (Adjuvant Chemotherapy[Title/Abstract])) OR (Adjuvant Drug Therapy[Title/Abstract]))) OR (chemotherapeutics[Title/Abstract]) |
| 6 | (((((((((((((((Peripheral Nervous System Diseases[Title/Abstract]) OR (Peripheral Nervous System Disease[Title/Abstract])) OR (PNS Diseases[Title/Abstract])) OR (PNS Disease[Title/Abstract])) OR (Peripheral Neuropathies[Title/Abstract])) OR (Neuropathy, Peripheral[Title/Abstract])) OR (Peripheral Neuropathy[Title/Abstract])) OR (Peripheral Nerve Diseases[Title/Abstract])) OR (Nerve Disease, Peripheral[Title/Abstract])) OR (Nerve Diseases, Peripheral[Title/Abstract])) OR (Peripheral Nerve Disease[Title/Abstract])) OR (Peripheral Nervous System Disorders[Title/Abstract])) OR (peripheral nerve disorder[Title/Abstract])) OR (peripheral nerve injuries[Title/Abstract])) OR (peripheral nervous disease[Title/Abstract])) OR (Polyneuropathy[Title/Abstract]) |
| 5 | chemotherapeutics[Title/Abstract] |
| 4 | ((((Chemotherapy, Adjuvant[Title/Abstract]) OR (Chemotherapy[Title/Abstract])) OR (Drug Therapy, Adjuvant[Title/Abstract])) OR (Adjuvant Chemotherapy[Title/Abstract])) OR (Adjuvant Drug Therapy[Title/Abstract]) |
| 3 | ("Randomized Controlled Trial" [Publication Type]) OR "Randomized Controlled Trials as Topic"[Mesh] |
| 2 | "Peripheral Nervous System Diseases"[Mesh] |
| 1 | "Chemotherapy, Adjuvant"[Mesh] |

| **EMBASE:** | |
| --- | --- |
| #16 | #14 AND #15 |
| #15 | #5 OR #8 |
| #14 | #12 OR #13 |
| #13 | #10 AND #11 |
| #12 | #2 OR #9 |
| #11 | #4 OR #7 |
| #10 | #3 OR #6 |
| #9 | 'chemotherapy-induced peripheral neuropathy':ab,ti OR 'chemotherapy neuropathy':ab,ti OR 'chemotherapy adj3 neuropathy':ab,ti |
| #8 | 'randomized controlled trials as topic':ab,ti OR 'randomized controlled trial':ab,ti OR 'clinical trials, randomized':ab,ti OR 'trials, randomized clinical':ab,ti OR 'controlled clinical trials, randomized':ab,ti OR 'pragmatic clinical trials as topic':ab,ti OR randomized:ab,ti OR 'controlled clinical trial':ab,ti OR randomly:ab,ti OR blind:ab,ti |
| #7 | 'peripheral nervous system diseases':ab,ti OR 'peripheral nervous system disease':ab,ti OR 'pns diseases':ab,ti OR 'pns disease':ab,ti OR 'peripheral neuropathies':ab,ti OR 'neuropathy, peripheral':ab,ti OR 'peripheral neuropathy':ab,ti OR (pns:ab,ti AND 'peripheral nervous system':ab,ti AND diseases:ab,ti) OR 'peripheral nerve diseases':ab,ti OR 'nerve disease, peripheral':ab,ti OR 'nerve diseases, peripheral':ab,ti OR 'peripheral nerve disease':ab,ti OR 'peripheral nervous system disorders':ab,ti OR 'peripheral nerve disorder':ab,ti OR 'peripheral nerve injuries':ab,ti OR 'peripheral nervous disease':ab,ti OR polyneuropathy:ab,ti |
| #6 | chemotherapy:ab,ti OR 'chemotherapy, adjuvant':ab,ti OR 'drug therapy, adjuvant':ab,ti OR 'adjuvant chemotherapy':ab,ti OR 'adjuvant drug therapy':ab,ti OR chemotherapeutics:ab,ti |
| #5 | 'randomized controlled trial'/exp OR 'randomized controlled trial (topic)'/exp |
| #4 | 'peripheral neuropathy'/exp |
| #3 | 'chemotherapy'/exp |
| #2 | 'chemotherapy-induced peripheral neuropathy'/exp |
| #1 | 'chemotherapy-induced peripheral neuropathy'/exp OR 'chemotherapy-induced peripheral neuropathy' |

| Cochrane Library | |
| --- | --- |
| #1 | MeSH descriptor: [Chemotherapy, Adjuvant] explode all trees |
| #2 | (Chemotherapy, Adjuvant):ti,ab,kw OR (Chemotherapy):ti,ab,kw OR (Drug Therapy, Adjuvant):ti,ab,kw OR (Adjuvant Chemotherapy):ti,ab,kw OR (Adjuvant Drug Therapy):ti,ab,kw |
| #3 | (chemotherapeutics):ti,ab,kw |
| #4 | #1 or #2 or #3 |
| #5 | MeSH descriptor: [Peripheral Nervous System Diseases] explode all trees |
| #6 | (Peripheral Nervous System Diseases):ti,ab,kw OR (Peripheral Nervous System Disease):ti,ab,kw OR (PNS Diseases):ti,ab,kw OR (PNS Disease):ti,ab,kw OR (Peripheral Neuropathies):ti,ab,kw |
| #7 | (Neuropathy, Peripheral):ti,ab,kw OR (Peripheral Neuropathy):ti,ab,kw OR (PNS (Peripheral Nervous System) Diseases):ti,ab,kw OR (Peripheral Nerve Diseases):ti,ab,kw OR (Nerve Disease, Peripheral):ti,ab,kw |
| #8 | (Nerve Diseases, Peripheral):ti,ab,kw OR (Peripheral Nerve Disease):ti,ab,kw OR (Peripheral Nervous System Disorders):ti,ab,kw OR (peripheral nerve disorder):ti,ab,kw OR (peripheral nerve injuries):ti,ab,kw |
| #9 | (peripheral nervous disease):ti,ab,kw OR (Polyneuropathy):ti,ab,kw |
| #10 | #5 or #6 or #7 or #8 or #9 |
| #11 | (chemotherapy-induced peripheral neuropathy):ti,ab,kw OR (chemotherapy neuropathy):ti,ab,kw OR (chemotherapy adj3 neuropathy):ti,ab,kw |
| #12 | #4 and #10 |
| #13 | #12 or #11 |
| #14 | MeSH descriptor: [Randomized Controlled Trials as Topic] explode all trees |
| #15 | MeSH descriptor: [Randomized Controlled Trial] explode all trees |
| #16 | (Randomized Controlled Trials as Topic):ti,ab,kw OR (randomized controlled trial):ti,ab,kw OR (Clinical Trials, Randomized):ti,ab,kw OR (Trials, Randomized Clinical):ti,ab,kw OR (Controlled Clinical Trials, Randomized):ti,ab,kw |
| #17 | (pragmatic clinical trials as topic):ti,ab,kw OR (controlled clinical trial):ti,ab,kw OR (randomized):ti,ab,kw OR (randomly):ti,ab,kw OR (blind):ti,ab,kw |
| #18 | #14 or #15 or #16 or #17 |
| #19 | #13 and #18 |

| WOS | | |
| --- | --- | --- |
| 1 | Chemotherapy, Adjuvant (topic) or Chemotherapy (topic) or Drug Therapy, Adjuvant (topic) or Adjuvant Chemotherapy (topic) or Adjuvant Drug Therapy (topic) or chemotherapeutics (topic) | TS=(Chemotherapy, Adjuvant) OR TS=(Chemotherapy) OR TS=(Drug Therapy, Adjuvant) OR TS=(Adjuvant Chemotherapy) OR TS=(Adjuvant Drug Therapy) OR TS=(chemotherapeutics) |
| 2 | Peripheral Nervous System Diseases (topic) or Peripheral Nervous System Disease (topic) or PNS Diseases (topic) or PNS Disease (topic) or Peripheral Neuropathies (topic) or Neuropathy, Peripheral (topic) or Peripheral Neuropathy (topic) or PNS (Peripheral Nervous System) Diseases (topic) or Peripheral Nerve Diseases (topic) or Nerve Disease, Peripheral (topic) or Nerve Diseases, Peripheral (topic) or Peripheral Nerve Disease (topic) or Peripheral Nervous System Disorders (topic) or peripheral nerve disorder (topic) or peripheral nerve injuries (topic) or peripheral nervous disease (topic) or Polyneuropathy (topic) | TS=(Peripheral Nervous System Diseases) OR TS=(Peripheral Nervous System Disease) OR TS=(PNS Diseases) OR TS=(PNS Disease) OR TS=(Peripheral Neuropathies) OR TS=(Neuropathy, Peripheral) OR TS=(Peripheral Neuropathy) OR TS=(PNS (Peripheral Nervous System) Diseases) OR TS=(Peripheral Nerve Diseases) OR TS=(Nerve Disease, Peripheral) OR TS=(Nerve Diseases, Peripheral) OR TS=(Peripheral Nerve Disease) OR TS=(Peripheral Nervous System Disorders) OR TS=(peripheral nerve disorder) OR TS=(peripheral nerve injuries) OR TS=(peripheral nervous disease) OR TS=(Polyneuropathy) |
| 3 | chemotherapy-induced peripheral neuropathy (topic) or chemotherapy neuropathy (topic) or chemotherapy adj3 neuropathy (topic) | TS=(chemotherapy-induced peripheral neuropathy) OR TS=(chemotherapy neuropathy) OR TS=(chemotherapy adj3 neuropathy) |
| 4 | Randomized Controlled Trials as Topic (topic) or randomized controlled trial (topic) or Clinical Trials, Randomized (topic) or Trials, Randomized Clinical (topic) or Controlled Clinical Trials, Randomized (topic) or pragmatic clinical trials as topic (topic) or controlled clinical trial (topic) or randomized (topic) or randomly (topic) or blind (topic) | TS=(Randomized Controlled Trials as Topic) OR TS=(randomized controlled trial) OR TS=(Clinical Trials, Randomized) OR TS=(Trials, Randomized Clinical) OR TS=(Controlled Clinical Trials, Randomized) OR TS=(pragmatic clinical trials as topic) OR TS=(controlled clinical trial) OR TS=(randomized) OR TS=(randomly) OR TS=(blind) |
| 5 | #1 AND #2 |  |
| 6 | #5 OR #3 |  |
| 7 | #4 AND #6 |  |
