# Supplementary material for: Non-pharmacological therapy for chemotherapy-induced peripheral neurotoxicity: a network meta-analysis of randomized controlled trials
Source: BMC Neurol. 2023 Dec 11;23:433. doi: 10.1186/s12883-023-03485-z (PMC10712106; doi:10.1186/s12883-023-03485-z)
Supplement: Supplementary file 2 — Supplementary Material 2 [file 12883_2023_3485_MOESM2_ESM.docx]

Supplementary material 2: Study characteristics

| **Study** | **Country** | **Cancer Type** | **Chemotherapy Drugs** | **Sample size** | **Male/Female** | **Age** | **interventions** | **Follow-up Time** | **Outcomes** |
| --- | --- | --- | --- | --- | --- | --- | --- | --- | --- |
| Song2020^(12)^ | Korea | Breast cancer | NA | 72 | 0/72 | 49.1 ± 8.85 49.71 ± 8.24 | Electrostimulation Sham-Electrostimulation | 2 weeks | CIPN symptoms Quality of Life Adverse Event |
| Zimmer2018^(29)^ | Germany | Stage IV colorectal cancer | FOLFIRI ± antibody FOLFOX ± antibody 5-fu/folinic acid ± antibody Capecitabine + antibody | 30 | 21/9 | 68.53 ± 7.75 70.00 ± 7.75 | Multimodal Exercise Usual care | 12 weeks | CIPN symptoms Balance  Strength Endurance capacity |
| Bao2020^(13)^ | USA | Breast Uterine  Ovarian | Carboplatin Docetaxel Docetaxel + Carboplatin Paclitaxel Paclitaxel + Carboplatin | 41 | 0/41 | 61.7 ± 10.88 | Yoga  Usual care | 12 weeks | CIPN symptoms Quality of life Balance Fall Gait speed  Adverse Events |
| Kim2021^(36)^ | Korea | Breast cancer | NA | 58 | 0/58 | 52.1 | Self-acupressure Usual care | 3 weeks | CIPN symptoms Quality of life |
| Streckmann2019^(27)^ | Germany | Mamma cancer Ovarial Adeno Colon Pancreatic Tcell NHL M. Hodgkin Plasmocytoma  Multiple myeloma Rectal Lung | Taxane Platinum derivate  Vinca alkaloid | 40 | 13/27 | 56 ± 6.75 | Sensorimotor training Vibration training  Oncological control group Healthy control | 6 weeks | CIPN symptoms Nerve conduction velocity Balance control Quality of life Pain |
| Smith2020^(14)^ | USA | Colorectal cancer Mreast cancer Myeloma | NA | 35 | 9/26 | 59 ± 9 | Scrambler therapy Sham control | 12 weeks | Pain CIPN symptoms |
| Shigematsu2020^(28)^ | Japan | Breast cancer | Paclitaxel | 44 | 0/44 | ≥66, 11 ＜77, 33 | Cryotherapy Usual care | 12 weeks | CIPN symptoms Tolerability of cryotherapy Adverse Event Compliance to cryotherapy |
| Schönsteiner2017^(42)^ | Germany | Multiple myeloma  Lymphomas Leukemias Colorectal Lung Esophageal/Gastric  Breast/Ovarian Other | Platinum‑basedm Taxane‑based Vinca alkaloids Bortezomib | 131 | 63/68 | 59 ± 10.5 62 ± 11.75 | Massage and passive mobilization+WBV Massage and passive mobilization | 19 weeks | CIPN symptoms Quality of life Physical examination Chair‑rising test Quantitative sensory testing |
| Saraboon2021^(45)^ | Thailand | Ovary Cervix | Paclitaxel + Carboplatin  Paclitaxel + Cisplatin | 30 | 0/30 | 45.53 ± 4.64 45.07 ± 3.88 | Balance exercise Usual care | 6 weeks | Balance Physical performance CIPN symptoms Quality of life |
| Rostock2013^(37)^ | Germany | Breast cancer  Ovarian cancer  Other Lymphoma | Vinca alkaloids Platin derivatives alone  Taxanes alone Platin derivatives and taxanes combined  Total no. of different cytostatics No.of diff.neurotoxic cytostatics only | 59 | 13/46 | 52.7 ± 10.0 | Electroacupuncture Hydroelectric baths  Vitamin B1 and B6  Placebo | 12weeks | CIPN symptoms Electroneurographical tests Quality of life |
| Prinsloo2018^(15)^ | USA | Breast Gastrointestinal  Gynecological Other | Paclitaxel Oxaliplatin Other taxane  Other platinum Taxane + platinum  Other | 71 | 9/62 | 62.5 ± 10.3 | Neurofeedback Usual care | 26 weeks | Pain Cancer-related symptoms Quality of life Sleep Fatigue |
| Knoerl2022^(16)^ | USA | Stage I–IV  Breast Colorectal Gynecologic | Oxaliplatin Taxanes Taxanes + Platinums | 44 | 2/42 | 60 ± 10.25 56.5 ± 9.75 | Yoga Usual care | 8 weeks | Pain Sleep Anxiety Fatigue Depression CIPN symptoms |
| Kurt2018^(17)^ | Turkey | Breast Digestive system cancers  Other Cancer | Eloxatin-based Taxan-based  Platin-based Taxan-Platin-based  Fluoracil-based | 60 | 32/28 | 58.33 ± 11.24 57.86 ± 10.56 | Reflexology massage Usual care | 6 weeks | CIPN symptoms Pain |
| Knoerl2018^(18)^ | USA | Breast Gastrointestinal  Genitourinary Lung  Multiple Lymphoma | Platinums Taxanes Bortezomib Vinca alkaloids  Multiple | 60 | 15/45 | 58.93 ± 9.33 63.37 ± 8.36 | Cognitive pain management Usual care | 8 weeks | Pain CIPN symptoms Comorbid symptoms |
| Joy2022^(19)^ | Belgium | Breast | Exposure Paclitaxel Paclitaxel and carboplatin Docetaxel | 32 | 0/32 | 49.75 ± 11.25 51.88 ± 11.31 | Photobiomodulation Placebo treatments | 15-21 weeks | Neuropathy Score Quality of life Aerobic capacity and endurance Pain |
| Onazi2021^(30)^ | Canada | Colon Colorectal  Rectal | Oxaliplatin | 31 | 12/19 | 60.1 ± 10.75 | Ultrasound+ home exercise  Exercise | 6 weeks | Pain Quality of life Protective Sensation Balance Reflexes |
| Müller2021^(38)^ | Germany | Breast Pancreatic cancer Prostate Stomach Oesophagus  Colon  Brain cancer Ovary  Tongue base cancer Rectal Other | Taxane-based Taxane-platinum combination Platinum-based  Vinca alkaloid  Platinum-vinca alkaloid combination | 163 | 25/138 | 53.3 ± 11.5 | Sensorimotor Resistance training Usual care | 44 weeks | CIPN symptoms  Balance Muscle strength  Quality of life  Adverse events |
| Molassiotis2019^(31)^ | China | Ovarian Head and neck  Breast Colorectal  Myeloma | Oxaliplatin Carboplatin Cisplatin Paclitaxel Docetaxel Capecitabine Bortezomib | 87 | 24/63 | 57.1 ± 7.7 | Acupuncture Usual care | 20 weeks | Pain Adverse Events Functional Assessment Nerve conduction study |
| Lu2020^(32)^ | USA | Breast cancer | Taxane | 40 | 0/40 | 54 ± 9 53.5 ± 11.25 | Acupuncture Usual care | 16 weeks | CIPN symptoms Pain Quality of Life |
| Knoerl2018(1)^(20)^ | USA | Breast Head and Neck  Colorectal  Prostate Esophageal  Testicular Gastrointestinal  Sarcoma  Bladder  Gastric Pancreatic Unknown Primary | Taxane  Platinum  Platinum +Taxane | 220 | 102/118 | 54 ± 15 56 ± 16.25 | Electronic Symptom Assessment-Cancer (ESRA-C) Usual education | 3–6 weeks | Physical function Pain  CIPN symptoms Depression Fatigue Insomnia |
| Kneis2019^(33)^ | Germany | Breast cancer Colorectal cancer Gynecological cancer other than breast Upper gastrointestinal cancerNon-small cell lung cancer Non-Hodgkin’s lymphoma  Multiple myeloma | NA | 41 | 11/30 | 62 ± 9.5 | Endurance+ Balance training Endurance training | 12 weeks | Functional performance Cardiorespiratory fitness Vibration sense CIPN symptoms |
| Iravani2020^(22)^ | China，Iran | Breast cancer Lung cancer Ovarian cancer Prostate cancer Colorectal cancer | Taxane Platinum compound Platinum compound-taxane Doxorubicin/Cyclophosphamide-taxane | 38 | 15/23 | 57.95 ± 10.39 58.79 ± 8.36 | Acupuncture Vit B1+ Gabapentin Group | 4 weeks/8 weeks | CIPN symptom Nerve conduction study Overall satisfaction Safety |
| Huang2021^(43)^ | China | Breast cancer | Docetaxel Carboplatin Other taxanes Other platinum | 20 | 0/20 | 49.60 ± 11.13 | Acupuncture  Sham acupuncture  Usual care | 9 weeks | Quantitative tactile detection thresholds Quality of Life CIPN symptoms Pain |
| Greenlee2016^(23)^ | USA | Stage I-III breast cancer | Paclitaxel only Paclitaxel and Docetaxel | 63 | 0/63 | 50 ± 11 | Electro-acupuncture sham EA | 16 weeks | Pain CIPN symptoms |
| Chitkumarn2022^(34)^ | Thailand | Ovary Endometrium Cervix Sarcoma | Paclitaxel  Cisplatin Carboplatin  Other (Ifosfamide) | 79 | 0/79 | 57.59 ± 11.23 55.45 ± 13.21 | Cold-therapy Usual care | 22 weeks | CIPN symptoms |
| Bao2020(1)^(21)^ | USA | Breast cance Colorectal cancer Other | Taxane-based only  Platinum-based only Taxane + platinum | 75 | 15/60 | 59.7 ± 12.4 | Acupuncture  Sham acupuncture  Usual care | 12 weeks | CIPN symptoms Pain |
| Bao2021^(35)^ | USA | Breast cance Colorectal cancer Other | Taxane-based only  Platinum-based only Taxane + platinum | 75 | 15/60 | 59.7 ± 12.4 | Acupuncture  Sham acupuncture  Usual care | 12 weeks | CIPN symptoms Anxiety and Depression Fatigue Pain Insomnia  Severity Index Quality of life |
| Noh2019^(39)^ | Korea | Ovarian cancer  Cervical cancer Endometrial cancer | Taxane + Platinium  Doxil (Caelyx)+ Platinum | 63 | 0/63 | 56.34 ± 9.04 55.36 ± 9.96 | Aroma self-foot reflexology training Usual care | 6 weeks | CIPN symptoms Peripheral skin temperature Anxiety and depression |
| Alessandro2022^(24)^ | Brazil | Breast  Gastrointestinal  Genitourinary Haematologic | NA | 29 | 12/17 | 57.68 | Acupuncture  Usual care | 7 weeks | CIPN symptoms Functional Independence Pain Quality of Life |
| Izgu2019^(25)^ | Turkey. | Colon cancer Rectal cancer  Gastric cancer | mFOLFOX-6 FOLFOX-6 | 46 | 27/19 | 56.45± 6.85 55.21± 9.99 | Aromatherapy massage Usual care | 8 weeks | Pain Severity of painful paresthesia Fatigue |
| Izgu2019(1)^(40)^ | Turkey. | Stage II-III breast cancer | Paclitaxel | 40 | 0/40 | 44.5 ± 10.7 47.0 ± 9.6 | Massage Usual care | 16 weeks | Pain Quality of life Nerve conduction studies |
| Ikio2022^(26)^ | Japan | Hematologic  Gastrointestinal cancer | Vincristine Oxaliplatin  Nab-paclitaxel | 39 | 22/17 | 69 ± 5.75 64 ± 7.5 | Exercise Usual care | 3-4 weeks | Upper-extremity function Strength Sensory function Manual dexterity Degree of symptoms Pain Quality of life |
| Bland2019^(46)^ | Canada | Stage I-III Breast cancer | Paclitaxel  Docetaxel | 27 | 0/27 | 50.2 ± 10.2 | Exercise Usual care | 18-27 weeks | CIPN symptomsand Quality of life Vibration sense and Pinprick |
| Lai2017^(44)^ | CHINA | Breast cancer | NA | 120 | 0/120 | 51.13 ± 9.02 | A nurse-led care program Usual care | 14-20 weeks | Quality of life Self-efficacy Symptom distress levels Satisfaction |
| Hammond2020^(41)^ | Canada | Stage I-III breast cancer | NA | 48 | 0/48 | 61.5 ± 10.25 | Physical therapy  Usual care | 24 weeks | Pain DASH Neuropathic Symptoms and Signs Quantitative sensory testing Activity |
| Lee2022^(47)^ | USA | Colon Rectal/colorectal  Pancreatic Esophageal | Oxaliplatin | 57 | 36/21 | 57.88±10.68 | Home-based brisk walking Physical activity education | 8 weeks | OIPN severity  Quality of life Physical and emotional function |
| Friedemann2022 | Germany | Breast cancer  Colorectal cancer  Pleura Mesothelioma  Testicular cancer  Tonsil cancer  Ovarian cancer  Lymphoma  Leucaemia  Glioblastoma  Endometrial cancer | Paclitaxel/ Docetaxel  Oxaliplatin / Cisplatin  Carboplatin + Paclitaxel  CHOP/BEACOPP  Melphalan  Temozolomide  Andriamycin  Cyclophosphamide | 60 | 9/51 | 61 ± 8.75  60.4 ± 8.25 | Acupuncture  Usual care | 14 weeks | Nerve conduction study  Clinical assessment  Patient-reported outcome measures (PROMs) |
| Stringer2022 | UK | Breast  Multiple Myeloma  Gastrointestinal  Gynaecological cancer | Platinum-based drugs  Taxanes  Thalidomide | 120 | NA | 61 ± 9.75  60 ± 12.5 | Acupuncture  Usual care | 10 weeks | Patient Reported Outcome Measure  CTCAE  Quality of life |
| Teng2022 | Australia | Breast  Colorectal  Other | Oxaliplatin  Docetaxel  Paclitaxel  Platinum and Taxane  Other | 44 | 17/27 | 61.8±9  61.7±11 | Photobiomodulation  Sham therapy | 12 weeks | CIPN symptoms  Quality of life |
| Cao2022 | USA | Ovarian cancer | NA | 134 | 0/134 | 57.2±9 57.9±7.8 | exercise Usual care | 1 year | CIPN severity |
| Chan2023 | China | StageⅢ-Ⅳcolorectal cancer | Oxaliplatin | 55 | 33/22 | 60.0±10.4 62.5±7.62 | Electroacupuncture Sham EA | 24 weeks | CIPN symptoms  Pain  Vibration sense test and light touch test  Quality of life |
| Gholamzadeh2023 | Iran | Colorectal cancer | NA | 80 | 43/37 | 56.8±11.3 55.5±9.7 | Reflexology Usual care | 4 weeks | Toxicity level  Quality of life |
| Huang2023 | China | Stage Ⅲ colorectal cancer | oxaliplatin | 20 | 10/16 | 52.0±4.13 52.0±4.25 | Acupuncture Sham acupuncture | 48 weeks | Nerve conduction velocity  Touch-detection thresholds  Quality of life  CIPN symptoms  Pain |
| Kanda2023 | Japan | Colorectal cancer | NA | 65 | 10/55 | 55.3±11.4 59.8±11.1 | Self-monitoring Usual care | 6 weeks | CIPN symptoms  Mental state  Symptom relief and safety behavior  Quality of life Self-efficacy |
| Jung2023 | Korea | Breast cancer | Taxane | 61 | 0/61 | 49.24±7.57 49.73±5.19 | Auricular acupressure  Usual care | 7 weeks | CIPN symptoms  Pain |
| Waibel2021 | Germany | Colorectal cancer Breast cancer Gynecological cancer Upper gastrointestinal cancer  Non-Hodgkin’s lymphoma | NA | 31 | 9/22 | 67 ± 9.5  60 ± 7.25 | balance+ moderate endurance training, moderate endurance training | 12 weeks | CIPN symptoms  Functional performance  Postural control behavior |
