# Supplementary material for: Non-pharmacological therapy for chemotherapy-induced peripheral neurotoxicity: a network meta-analysis of randomized controlled trials
Source: BMC Neurol. 2023 Dec 11;23:433. doi: 10.1186/s12883-023-03485-z (PMC10712106; doi:10.1186/s12883-023-03485-z)
Supplement: Supplementary file 3 — Supplementary Material 3 [file 12883_2023_3485_MOESM3_ESM.docx]

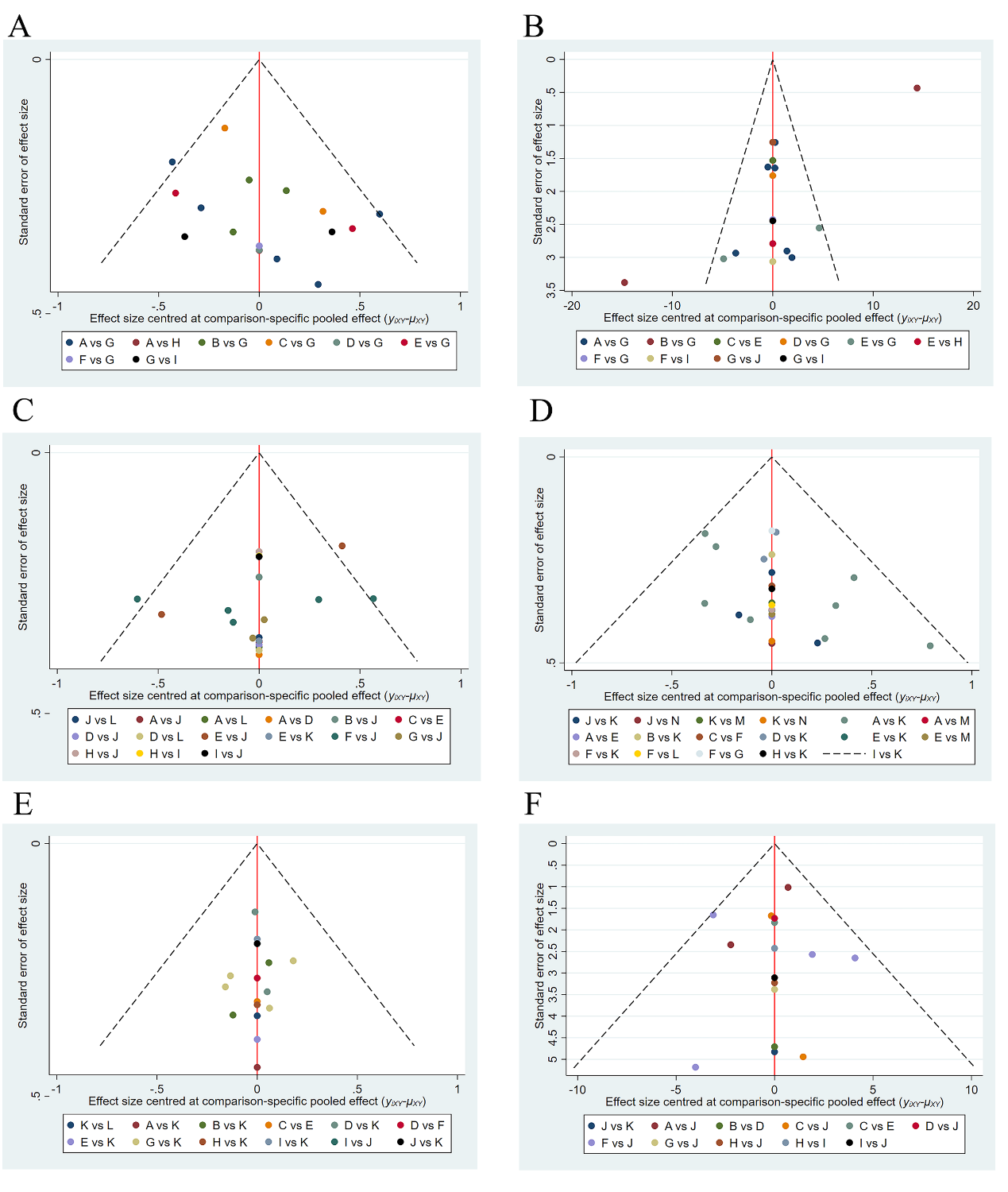


**Figure S1:** The funnel plot for inclusion studies: (A) Pain; (B) Peripheral neuropathological symptoms-FACT/GOG-Ntx; (C) Peripheral neuropathological symptoms-other scales; (D) Quality of life; (E) Sensory symptoms; (F) Motor symptoms.
